# Investigation of the effect of copper content added to aluminum on cutting force in MQL turning

**DOI:** 10.1038/s41598-025-34458-6

**Published:** 2026-01-13

**Authors:** Ferit Ficici, Ismail Ozdemir, Thomas Grund, Thomas Lampke

**Affiliations:** 1 GLOBAL ARGE, Gebze/Kocaeli, Gebze, Turkey; 2 Materials and Surface Engineering, Chemnitz University of Technology, Erfenschlager Str. 73, Chemnitz, 09125 Germany

**Keywords:** Aluminum casting, Metal cutting, MQL, Cutting force, Feed force, ANOVA, Regression, Tool wear, Engineering, Materials science

## Abstract

The purpose of this study is to investigate the influence of copper content and cutting parameters on the microstructure, hardness, machinability, and cutting-force behavior of sand-cast Al–Cu alloys under minimum quantity lubrication (MQL) conditions. In this study, workpieces obtained using the sand mold casting method were tested using the minimum quantity lubrication (MQL) method on a lathe. Five different test specimens were produced by adding copper to aluminum. The microstructures were examined via optical microscope, and hardness values were determined using Brinell hardness. Due to significant production and energy costs, the MQL method was preferred for metal cutting. Cutting forces generated during the process were measured using a dynamometer and analyzed in terms of feed rate, cutting velocity, and workpiece material using a full factorial analysis. Additionally, wear mechanisms on the cutting tool were determined using scanning electron microscope (SEM) images and Energy Dispersive Spectrum (EDS) analysis. The lowest hardness value was 34 HB in pure aluminum, while the highest was 95 HB in Al-8Cu alloy. Cutting forces increased with feed rate across all samples and decreased with higher cutting velocity. The highest cutting force (216.03 N) and feed force (26.90 N) were found in the Al-8Cu alloy, whereas the lowest cutting force (54 N) and feed force (6.07 N) were in pure aluminum. SEM and EDS analysis revealed flank wear and adhered aluminum on the cutting tools. Regression analysis verified that cutting velocity is the most critical factor influencing cutting and feed forces, with material type and feed rate also playing significant roles. Regression and ANOVA analyses consistently identified cutting velocity as the dominant factor affecting cutting and feed forces, followed by material type and feed rate.

## Introduction

 Aluminum is lightweight due to its low density; however, its low melting point and poor mechanical characteristics constrain its applications. Alloying aluminum with other elements can expand its range of applications and improve its properties. Additives to aluminum alloys include copper (Cu), silicon (Si), magnesium (Mg), manganese (Mn), zinc (Zn), titanium (Ti), iron (Fe) and others^1–4^.

Aluminum-copper alloys usually contain between 2% and 10% copper, with small additions of other elements. Copper provides aluminum with hardness, strength, castability and ease of machining. It also facilitates precipitation hardening. They are easy to prepare and cast. They have sufficient thermal conductivity and good heat resistance. Machinability is good and fluidity is less than silicon alloys^5–7^. The addition of copper to aluminum can also reduce ductility and corrosion resistance. Aluminum-copper alloys have increased susceptibility to solidification cracking. As a result, some of these alloys can be the most difficult aluminum alloys to weld. These alloys include some of the highest strength aluminum alloys that can be heat treated. The alloy is mostly cast in sand and the second alloy is cast in both sand and continuous molds. These alloys are used in aerospace, military vehicles, rocket blades, pistons and engine lugs^8–10^.

Machining is the most widely used method of manufacturing to create a variety of mechanical parts for industries such as automotive, aircraft, defense, and tool manufacturing. This procedure always includes high temperatures caused by friction and deformation of materials, therefore necessitating the use of lubricants. Since excessive heat is not desired, it can have a negative effect on the performance of both the cutting tool and the work-piece material being worked on^11,12^.

The process of machining aluminum results in a significant chip-to-tool contact area, accompanied by a high chip thickness ratio. This phenomenon leads to elevated cutting forces, increased machining power, and heightened heat generation. In contrast, the cutting forces encountered when machining aluminum alloys are typically lower than those associated with ferrous alloys, attributable to their reduced mechanical strength. Specific cutting pressures can be as much as 70% lower in aluminum machining compared to steel^13^. Nevertheless, it is important to recognize that this disparity is relatively minor among various aluminum alloys and is influenced by their specific chemical compositions and physical characteristics^14^. Any thermal or mechanical treatment, as well as the incorporation of alloying elements that enhance the hardness and mechanical strength of aluminum alloys, tends to diminish the chip-tool contact area. Consequently, this reduction can lead to a decrease in machining forces. This decrease effectively offsets the impacts of increased mechanical strength and the corresponding reduction in contact area^15,16^.

High temperatures in the machining zone during dry machining can result from higher order friction between the tool and the work as well as between the tool and the chip. In the finish, the high temperature at the machining zone will result in tool wear issues and dimensional errors for the work piece. Therefore, the disadvantages of dry machining must be reduced to gain it^17^.

Minimum quantity lubrication (MQL) can be utilized as an alternative to dry machining. The selection of a cutting fluid for Minimum Quantity Lubrication (MQL) should consider not only its primary attributes, particularly cutting performance, but also its secondary attributes, which include biodegradability, oxidation stability, and storage stability. Processes where friction and adhesion are predominant typically necessitate the use of minimal amounts of fluid. MQL denotes the application of minimal amounts of cutting fluids, typically three to four orders of magnitude less than those utilized in conventional flooded lubrication scenarios^18–20^. Cooling and/or cutting fluids are applied to the tool tip and workpiece interface to lower heat because of their lubricating properties and remove chips from the cutting zone. As a result, it increases dimensional accuracy and tool life in addition to product quality^21,22^. Factors affecting the cutting forces during machining of aluminum alloy include feed rate, cutting velocity and depth of cut. There are many studies in the literature on the machinability of aluminum alloys^23–27^.

Due to significant production and energy costs, the MQL method was preferred for metal cutting. While the environmental and economic advantages of MQL compared to conventional wet lubrication and dry machining are well recognized in the literature, this study focuses on MQL performance under selected machining conditions. Comparative data between dry, wet, and MQL machining have been reported elsewhere^28,29^, and the present work emphasizes the machinability behavior of Al–Cu alloys under MQL.

In this study, copper element was added to pure aluminum in different ratios and produced by sand mold casting method. The addition of copper element provided hardness, strength, castability and ease of processing of the test specimens. Copper was selected as the alloying element due to its well-documented contribution to hardness, strength, and machinability in Al–Cu alloys, which has been reported to be more favorable than Al–Si alloys in certain machining applications^30,31^. Finally, MQL was chosen because it offers a cost-effective production method using natural resources and minimizing the negative effects of cutting fluids.

## Materials and methods

The commercial purity Etial 8E alloy (0.10 wt.%Mg, 0.15 wt.%Fe, 0.03 wt.%Cu, 0.021 wt.%Mn, 0.009 wt% Mg and remaining wt% Al) used in the casting of the test specimens was obtained from Eti Alüminyum Seydişehir Factories as primary ingots. Cu alloy with 99.8% purity was used for Cu additions. After the alloys were prepared, the chemical analysis of the samples taken from the castings were determined by Spectromax X spectral analyzer (Spectro Analytical Instruments in Germany) to check the suitability of the chemical composition. The chemical analysis values obtained with the spectrometer for the alloys used are shown in Table 1.

**Table 1:** Chemical analysis values of the alloys (wt%).

Chemical Element (%)							
Alloy	Si	Fe	Cu	Mn		Mg	Al
Al	0.10	0.15	0.03	0.021		0.009	Balance
Al-2Cu	0.11	0.21	2.04	0.023		0.012	Balance
Al-4Cu	0.13	0.12	4.11	0.021		0.011	Balance
Al-6Cu	0.12	0.17	6.12	0.019		0.014	Balance
Al-8Cu	0.14	0.19	7.96	0.022		0.015	Balance

### Casting method

**Fig. 1:**
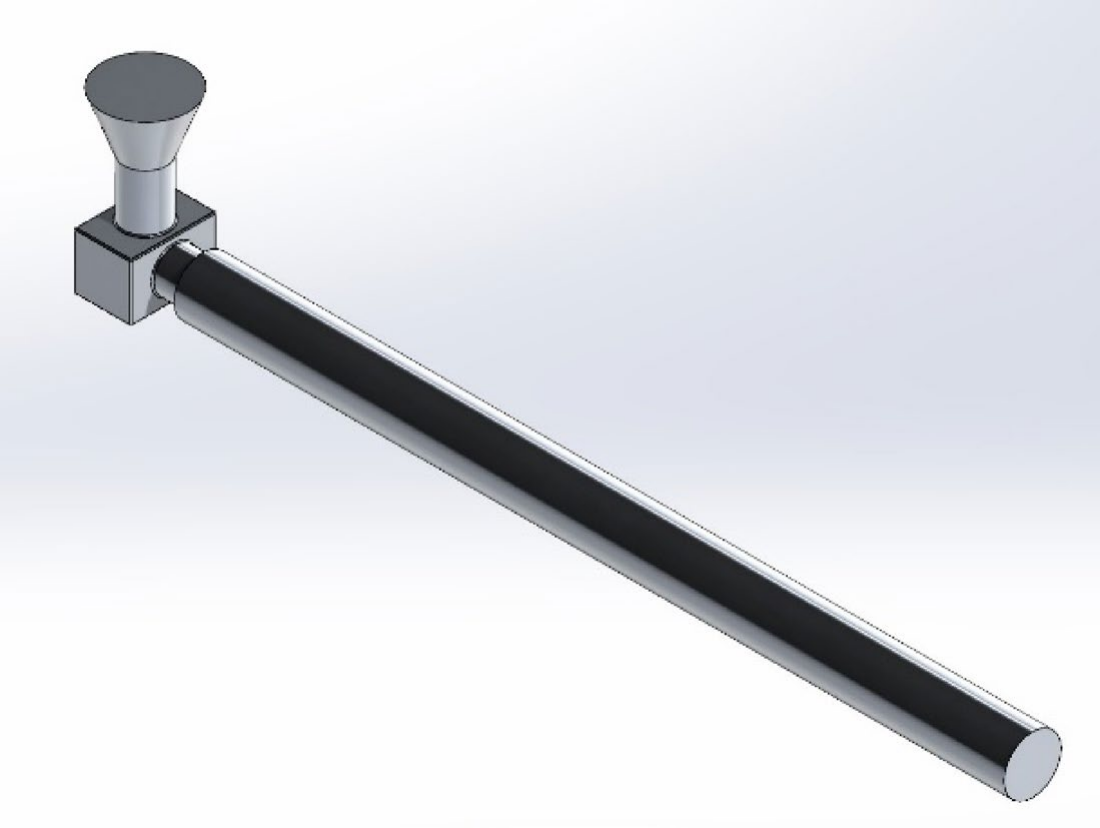
3D model produced from the sand mold.

In the experiments, pure aluminum and alloys containing 2 wt%, 4 wt%, 6 wt%, and 8 wt% Cu were produced. These alloys were prepared from commercial Etial 8E alloy with 99.8% purity. The melting processes were carried out in an 8 kg capacity electric resistance furnace with a carbide-based crucible. The test model was designed as a double-sided plate model and specially produced for ease of sand mold preparation. Female centering pins were placed in the lower degree and male centering pins in the upper degree to ensure the closure of the sand mold. Mold sand was obtained by adding 2.5% sodium silicate resin to dry silica sand with a grain size of 90–110 AFS. During mold preparation, graphite was sprinkled to prevent the mold sand from sticking to the model. Then the prepared mold sand was placed into the degree and compacted with a mallet. After the mold was hardened with gas, it could be easily separated from the model. The 3D model produced from the sand mold is shown in Fig. 1. The melting process was carried out in a 2-kW electric resistance furnace with a maximum temperature of 1000 °C and a SiC crucible. The furnace was heated to 750 °C to completely melt the aluminum alloy. To purify the molten metal from dissolved hydrogen gas, liquid nitrogen was applied at approximately 730 °C using a graphite lance. Then, the molten metal was covered with commercial powder flux and heated to 750 °C. The slag layer on the surface of the metal was cleaned and poured into the prepared mold.

### Experimental details

**Fig. 2:**
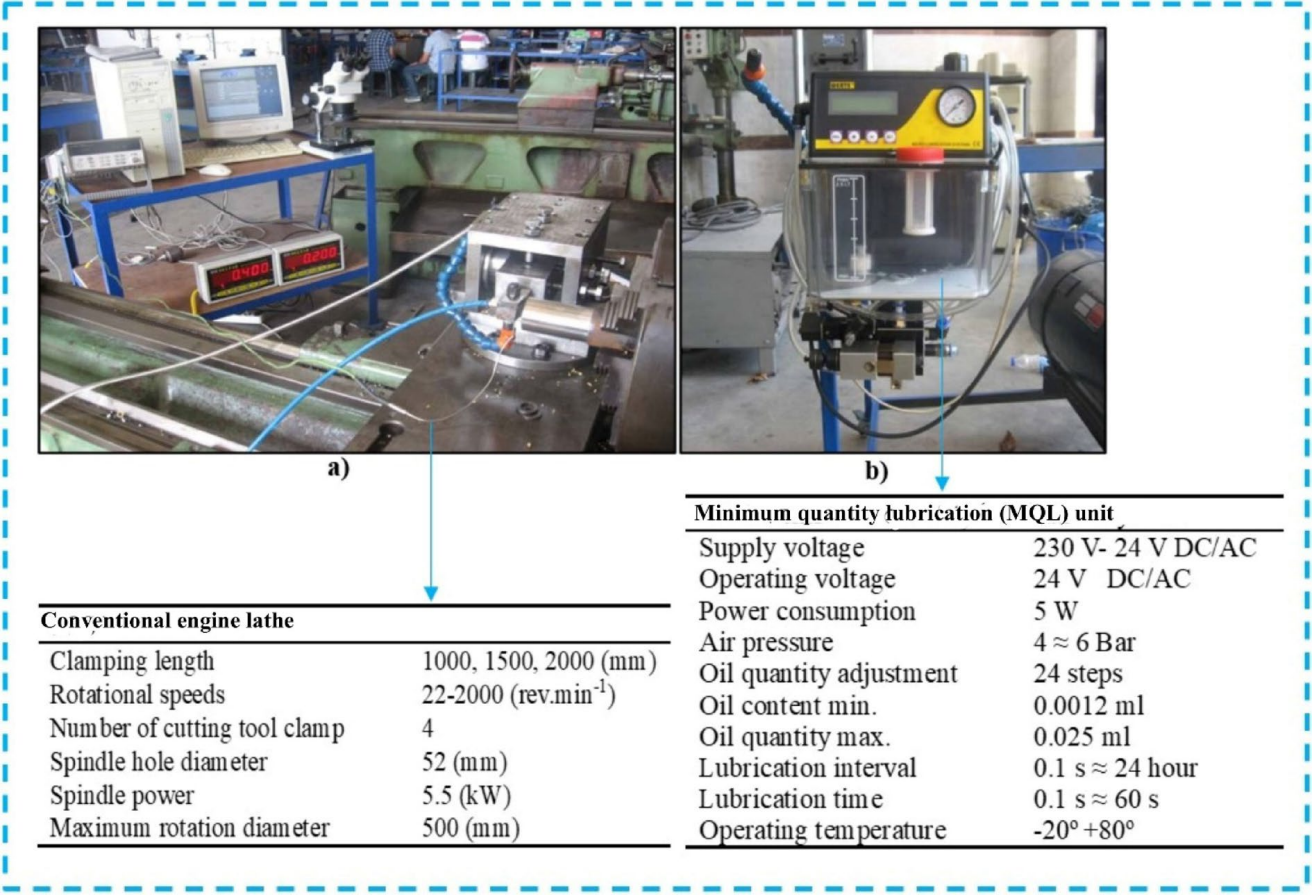
**(a)** Experimental-set-up, **(b)** minimum quantity lubrication (MQL) system.

For the cutting tests of the specimens obtained by casting method, **a conventional engine lathe was used**. Workability tests were carried out according to the test conditions in ISO 3685 standard. In addition, **a commercial minimum quantity lubrication (MQL) system** was preferred for cutting tests. The experimental set-up prepared for the cutting tests and their specifications are given in Fig. 2a, b. The lubricant application was determined at 100 ml/h. In this study, the advantages and limitations of applying MQL were evaluated based on several measurable criteria, including cutting forces, tool wear behavior, chip adhesion, and the thermal characteristics of the cutting zone. Lower cutting forces observed at higher cutting velocities, along with reduced flank wear and less built-up edge formation on the tool, were considered indicators of the benefits of MQL. Conversely, increased adhesion and flank wear at lower cutting velocities were regarded as limitations of the technique. These experimentally derived observations, supported by SEM and EDS anaopticlyses, provided a clear basis for assessing the overall performance of MQL under the selected machining conditions.

**Fig. 3:**
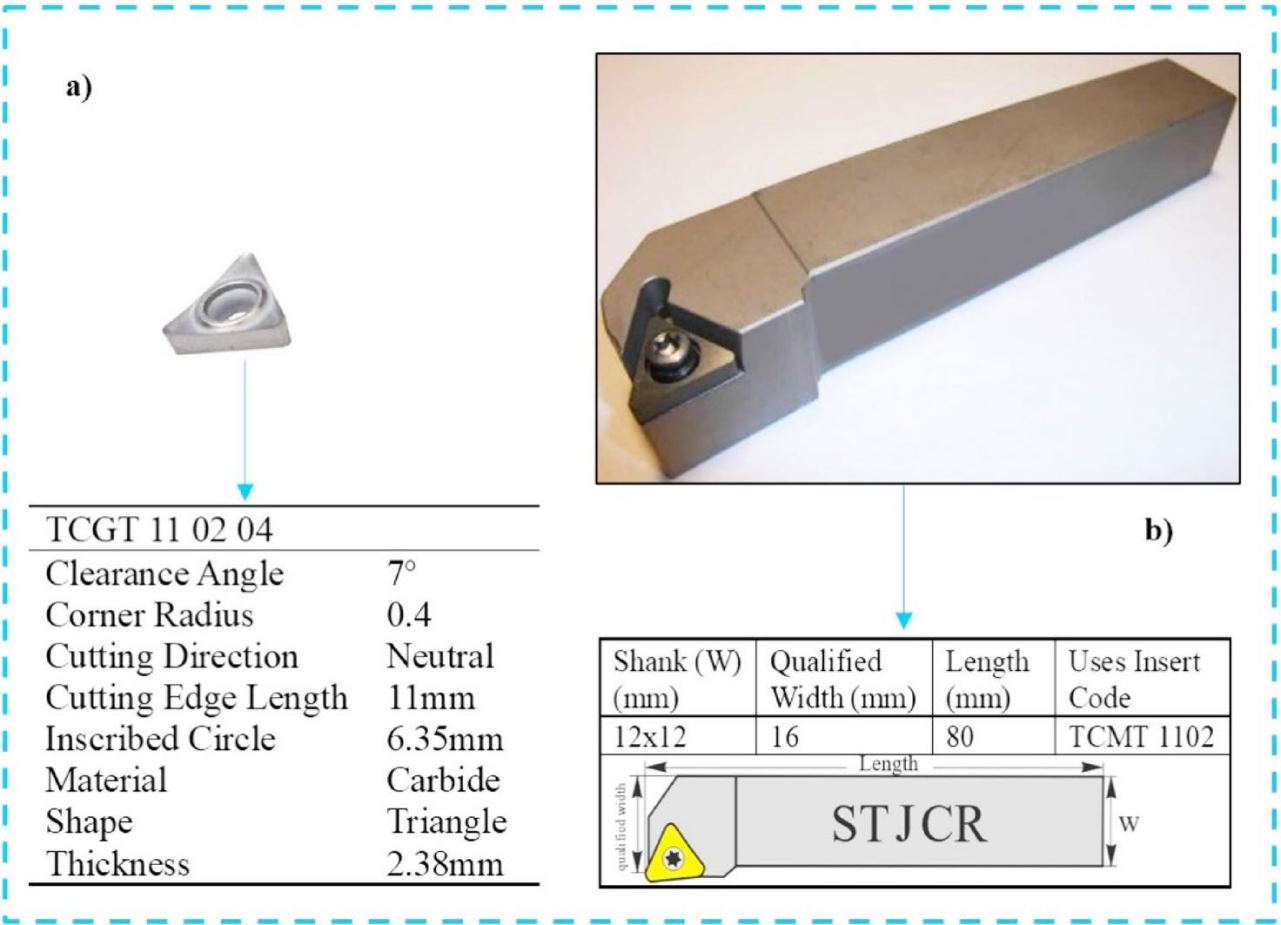
**(a)** Cutting tool, **(b)** Tool holder.

Uncoated k10 type carbide (polished) was used as cutting tool material. The tool insert type was selected as TCGT 11 02 04 in accordance with ISO standard. TCMT 11 02 coded in accordance with ISO standard was determined as the cutting tool holder. The pictures and characterizations of the cutting tool and holder are shown in Fig. 3a, b. A dynamometer was utilized to measure the cutting forces produced during the cutting process on the machine. Cutting forces were measured using a three-component piezoelectric dynamometer. The device provides a measurement capacity in the kilonewton range with high sensitivity suitable for detecting small dynamic variations in cutting forces, and the workpieces were rigidly mounted on the dynamometer’s top plate to ensure stable force transmission. Force signals were recorded using a high-speed data acquisition system at a sampling frequency of 20 kHz, which is sufficient to capture transient force fluctuations during turning. These specifications ensure accurate, reliable, and reproducible measurement of cutting forces throughout the experiments.

The test parameters required for the cutting tests were determined by considering the conditions recommended by the available literature and cutting tool catalogs. The test parameters and their levels applied in the cutting tests are given in Table 2. The cutting parameters in Table 2 were selected by considering the characteristics of the cast Al–Cu alloys and the limitations of the machining conditions. Due to the presence of Al–Cu intermetallic compounds, alloys with higher Cu content exhibited increased hardness and lower machinability, making reduced cutting velocities necessary to avoid excessive tool wear and instability. The use of MQL also limits the applicability of high cutting velocities, as they tend to increase thermal load and adhesion on the cutting edge, which is inconsistent with the recommendations for the uncoated K10 carbide insert used in this study. Therefore, lower cutting velocities ensured stable cutting behavior and reliable force measurements. Moreover, the selected cutting velocity range is consistent with previous studies on the MQL machining of cast aluminum alloys, which similarly recommend moderate cutting velocities to maintain tool life and cutting stability.

In this study, a full factorial design was implemented to systematically evaluate the effects of feed rate and cutting velocity on cutting forces. All combinations of the three feed rates (0.08, 0.16, and 0.32 mm/rev) and the three cutting velocities (30, 90, and 125 m/min) were tested. Accordingly, each cutting velocity was applied at all feed rates, and each feed rate was applied at all cutting velocities, rather than keeping one parameter constant. This design ensured a comprehensive and statistically reliable assessment of the independent and combined effects of these parameters on cutting force behavior.

**Table 2:** Test parameters and their values.

Test Parameter	Value	Unit
Depth of cut	1	mm
Feed rate	0.08–0.16.08.16-0.32	mm.rev^− 1^
Cutting velocity	30–90-125	m.min^− 1^

## Results and discussion

### Microstructure

**Fig. 4:**
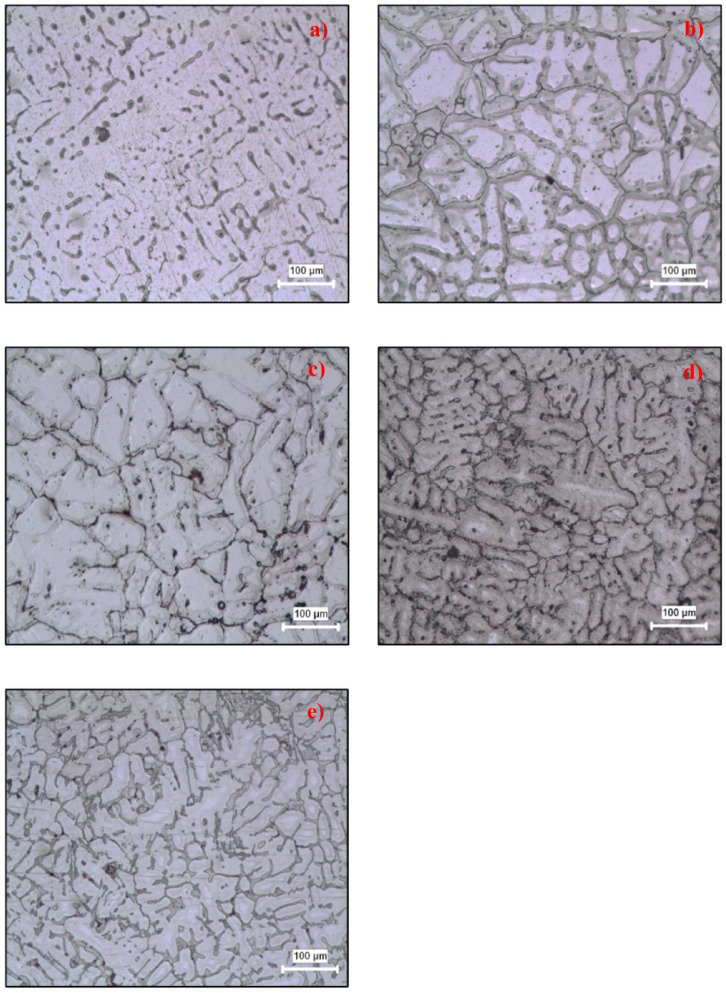
Microstructure of test materials **(a)** Al, **(b)** Al-2Cu, **(c)** Al-4Cu, **(d)** Al-6Cu, **(e)** Al-8Cu.

The samples that were cut from the castings were placed within bakelite for microstructure analyses. After rough and fine sanding with 60–180-400–800-1200-2500 grit sandpaper, the bakelite samples were polished with alumina suspension and etched with Keller solution and subjected to microstructure examinations. Microscopic examinations of the metallographically prepared surfaces were carried out using an optical light microscope and a digital camera coupled with an image analysis software. Figure 4 shows the microstructure images of the test specimens. Microstructure examinations were performed at 100x magnification.

According to the microstructure pictures, it was observed that the internal structures of the test specimens were solidified in various ways depending on the chemical composition. As determined in the microstructure examinations, the structure contains Al-Cu compounds in varying ratios depending on the α main matrix and Cu content. Accordingly, it is seen that more Al-Cu compounds are formed with increasing Cu content in the casting samples.

### Hardness

**Fig. 5:**
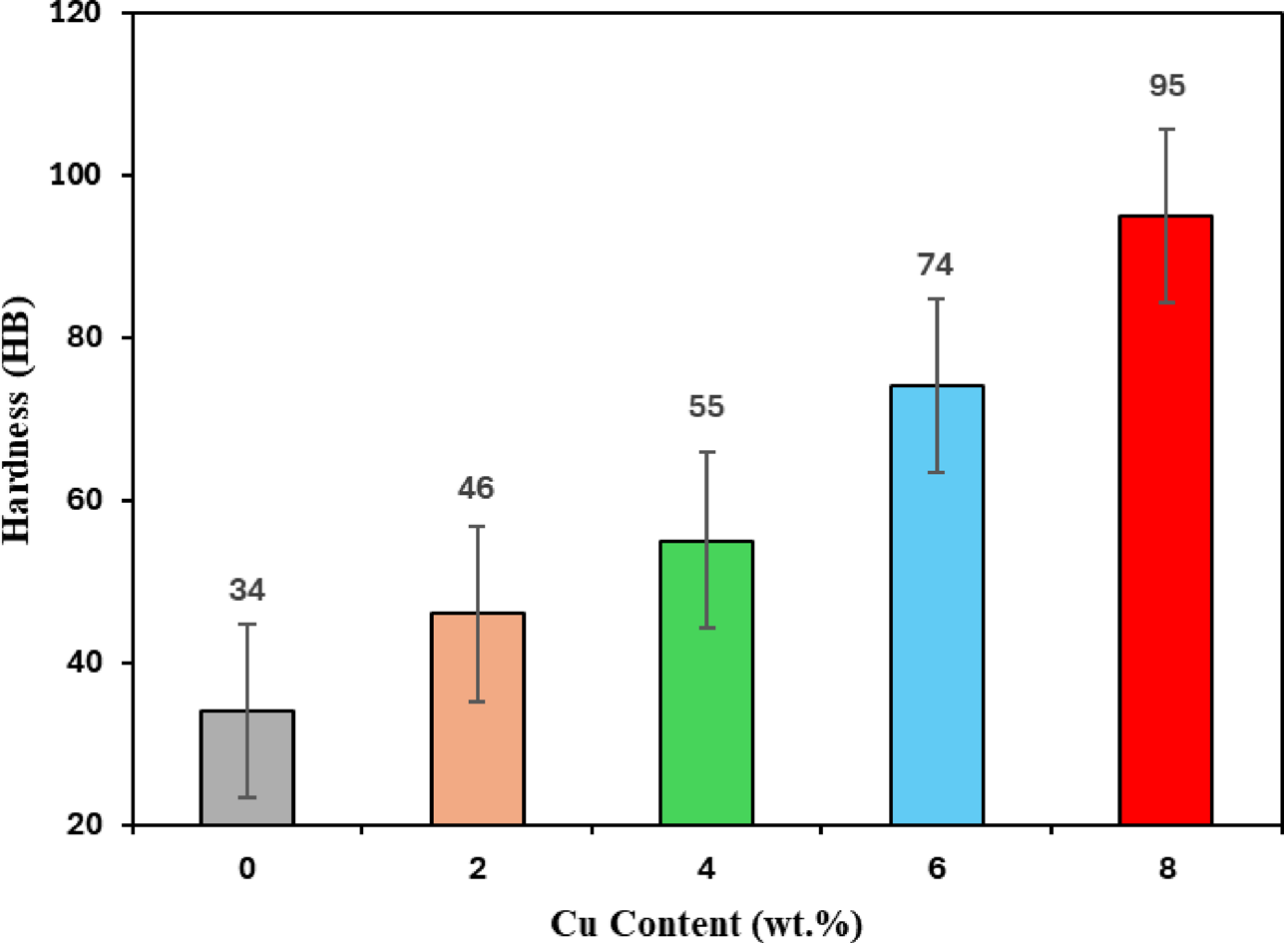
Hardness of test parameters.

To determine the hardness values of the cast samples, Brinell hardness measurements were made on a Brinell hardness testing machine. In the hardness tests, at least 3 hardness results of each sample were averaged under a 2.5 mm diameter ball and 62.5 kg load. The hardness values given in Table 4 are the average values of the samples taken for comparison purposes from the same areas of the alloys cast in the mold prepared using the same model. As can be seen from the hardness values given in Fig. 5, Cu content addition to Al alloys increases the hardness value. While the hardness value of pure Al is 34 HB, there is a hardness increase up to 3 times with 8% Cu content addition. An increase in the hardness value of the test specimens was observed with increasing Cu content addition. It was determined that the hardness increase was higher at high Cu content additions than at low Cu additions. The hardness increase with Cu content addition is thought to be due to the hard Al-Cu compounds formed in the structure with Cu content addition as seen in the microstructure pictures. Consequently, the same trends of Al-Cu variation should also be present in hardness variations. This result is in line with the experimental studies of Mehditabar et al.^32^.

The increase in cutting forces with higher Cu content is directly supported by both the microstructural observations and the mechanical property data presented in Fig. 4. As the Cu content increases, a larger amount of Al–Cu intermetallic precipitates forms within the aluminum matrix, which strengthens the alloy by restricting dislocation motion. This microstructural evolution is reflected in the substantial increase in hardness shown in Fig. 4, indicating a corresponding increase in yield and rupture strengths. These higher strength values require greater stress for chip initiation and deformation, explaining the higher cutting and feed forces observed in the Al–Cu-rich alloys. In addition, the thermal behavior of the cutting zone plays an important role: at higher cutting velocities, elevated temperatures reduce the material’s flow stress and promote localized softening, resulting in lower cutting forces. At lower cutting velocities, however, insufficient thermal softening maintains higher material strength and leads to increased force requirements. Together, the microstructural strengthening mechanisms, the hardness increase shown in Fig. 4, and the thermally influenced deformation behavior account for the cutting-force trends observed in this study.

### Cutting forces

In the cutting tests performed on five specimens obtained by casting method, the effects of feed rate, cutting velocity and copper content on the cutting forces were investigated.

#### Effect of feed rate

**Fig. 6:**
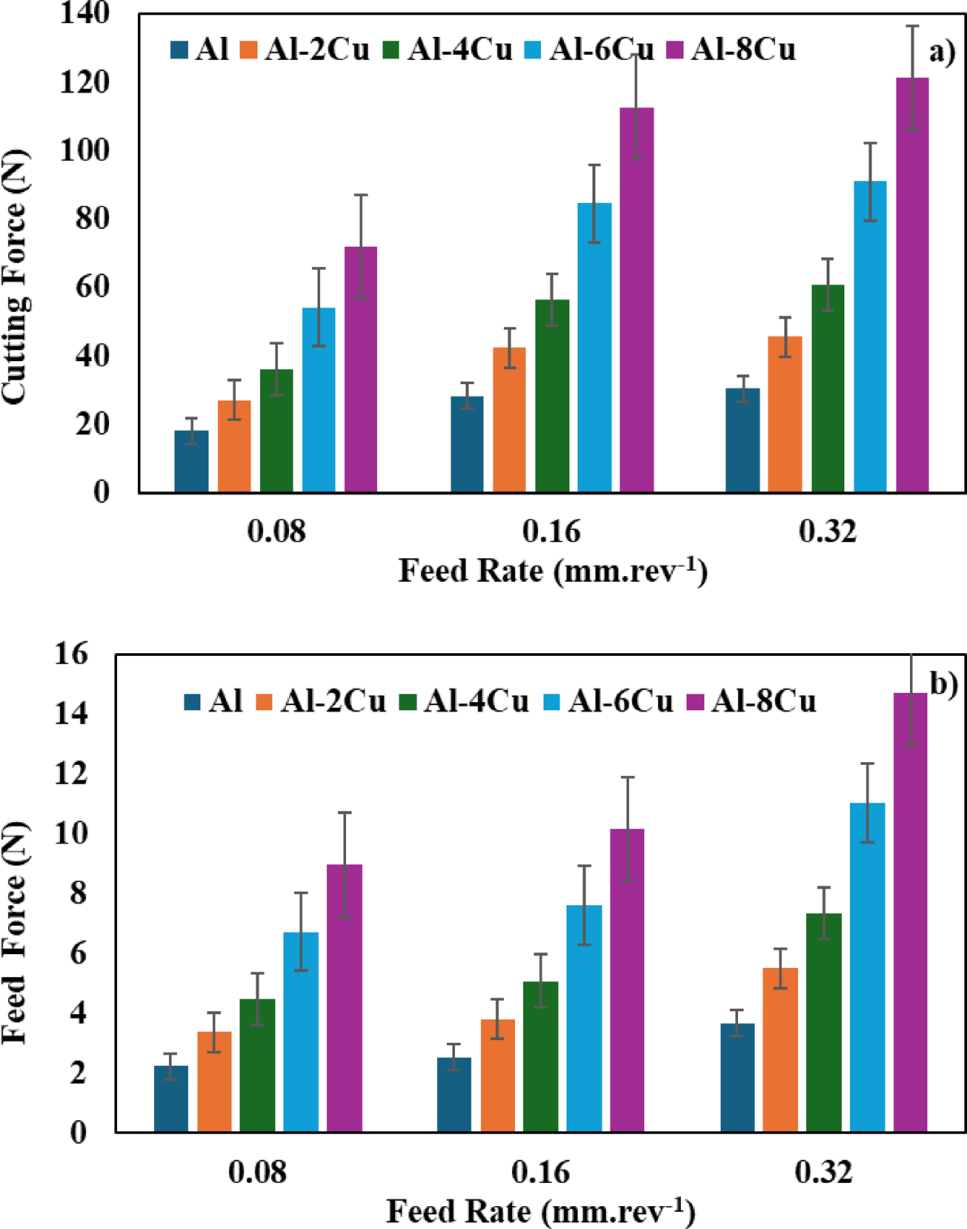
Effect of feed rate on **(a)** cutting force and **(b)** feed force.

The effect of feed rate change on cutting force and feed force is depicted in Fig. 6. When Fig. 6 is examined carefully, it is observed that an increase in cutting forces occurs with an increase in feed rate. Additionally, it was also found that the cutting forces exhibited similar behavior for all materials. The highest cutting force is generated at a feed rate of 0.32 mm/rev while the lowest cutting forces are generated at a feed rate of 0.08 mm/rev for both cutting force and feed force. This leads to an increase in feed rate, a significant increase in chip thickness and therefore higher cutting forces^33^. This also results in more volume being deformed and requiring additional cutting forces to cut the chip. Veera Ajay and Vinoth reported similar results in their optimization study on cutting forces, surface roughness and temperature of aluminum 6061 material using response surface methodology^34^.

#### Effect of cutting velocity

**Fig. 7:**
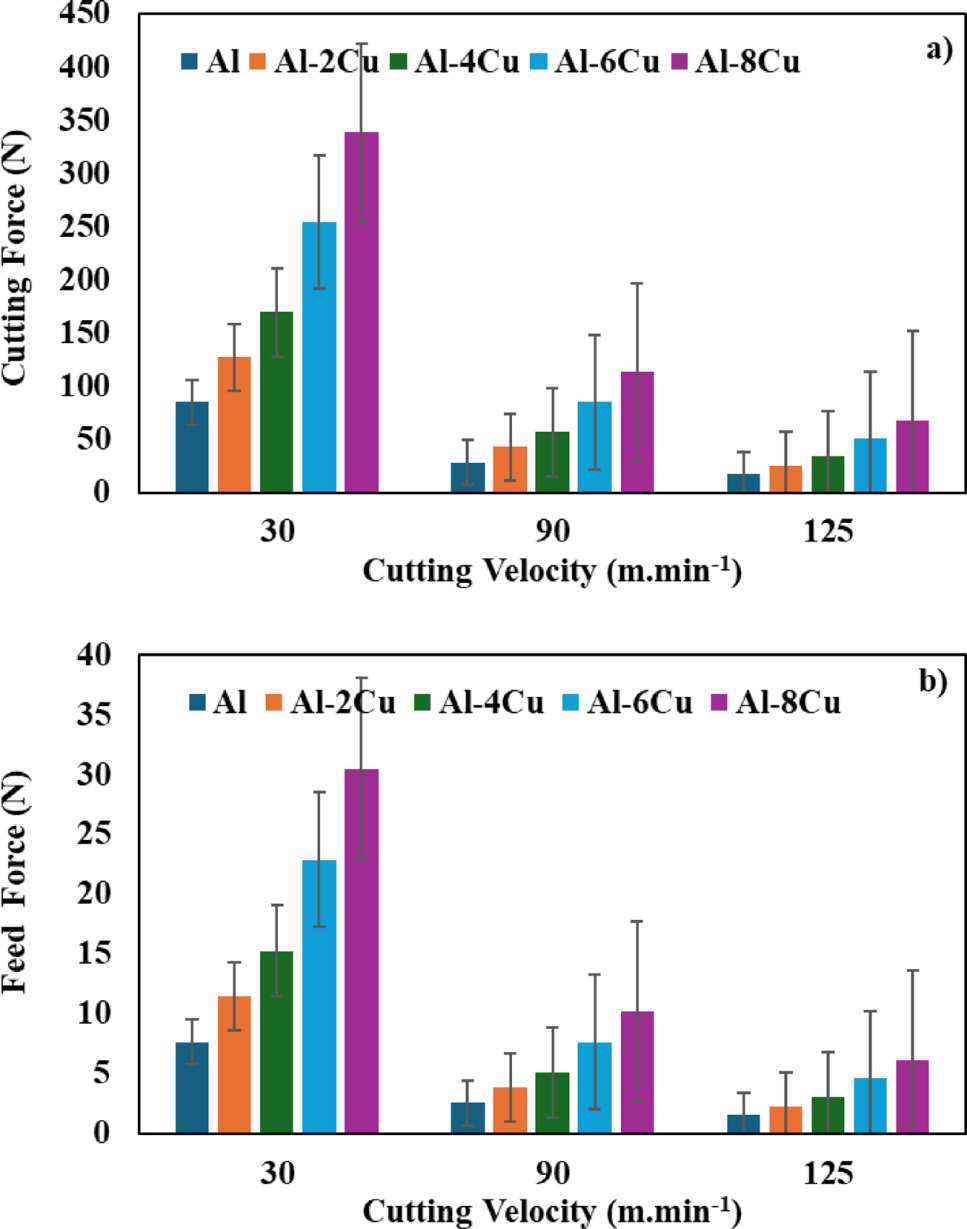
Effect of cutting velocity on **(a)** cutting force and **(b)** feed force.

The variation in cutting forces with increasing cutting velocity is depicted in Fig. 7. Based on Fig. 7, the cutting force (cutting force and feed force) decreased as the cutting velocity increased. The behavior was found to be consistent across all materials. This is because as the temperature in the cutting zone rises, the metal being machined becomes softer and requires less effort for turning. These findings are in good agreement with the literature that cutting forces increase under the MQL system^35–37^.

#### Effect of Cu content

**Fig. 8:**
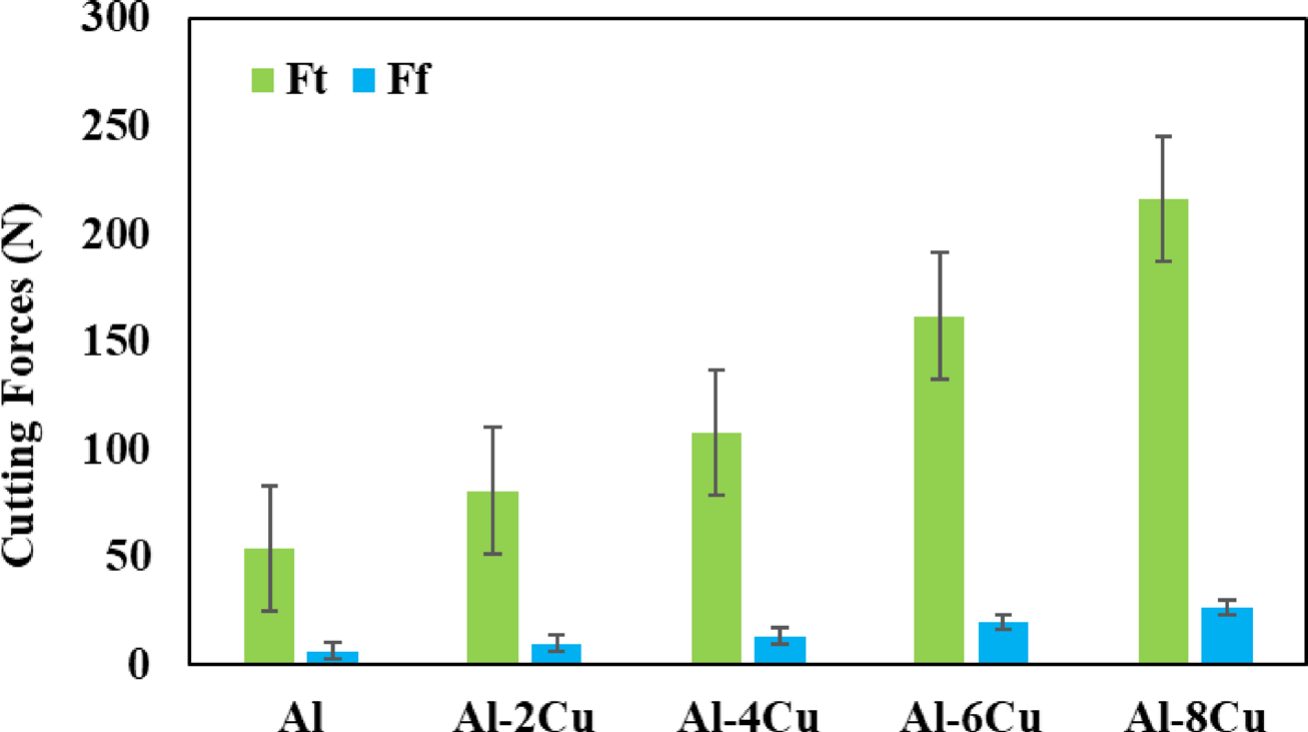
Effect of material type on cutting forces.

Figure 8 shows the effect of the test specimens obtained by casting method on the shear forces. When Fig. 8 is carefully examined, it is observed that the lowest cutting forces are obtained in the pure aluminum test specimen, while the highest cutting forces occur in the Al-8Cu test specimen. This suggests that there is a relationship between hardness and cutting force^38^. Accordingly, the Al specimen with the lowest hardness value produced the lowest cutting forces, while the Al-8 C specimen with the highest hardness value produced the highest cutting forces. Other studies have reported comparable findings^39,40^.

### Wear mechanism of cutting tool

**Fig. 9:**
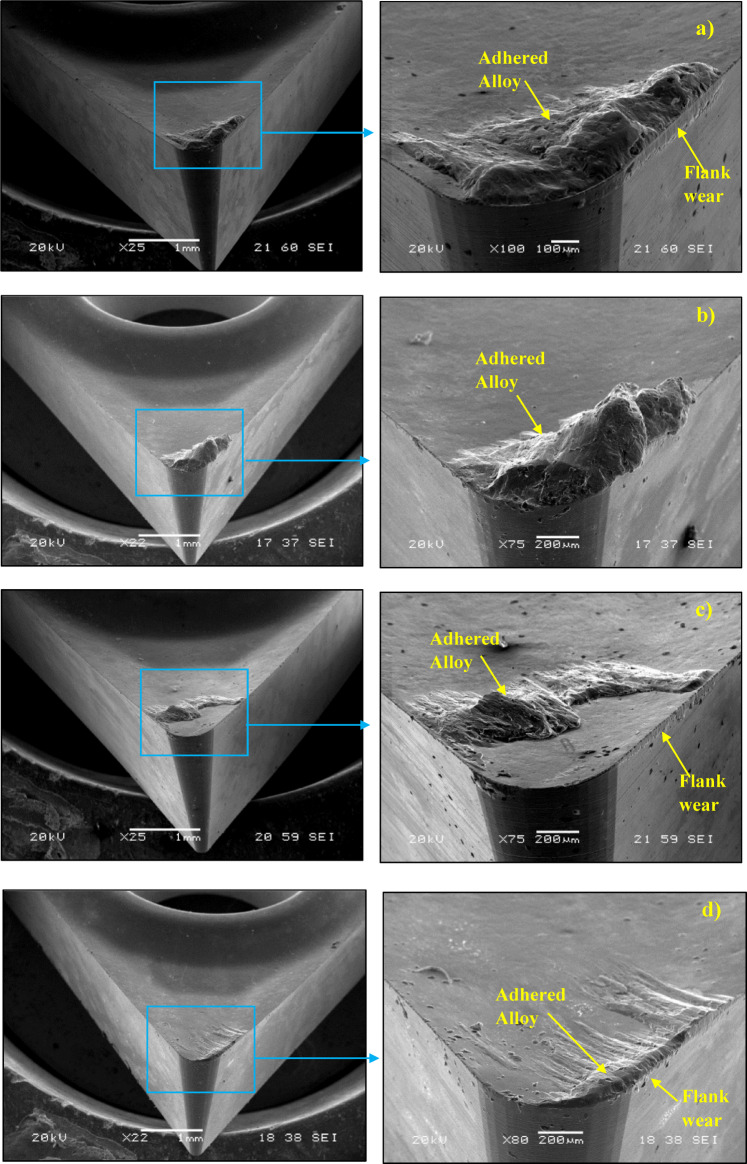
SEM images of test materials at 0.08 (mm*rev^− 1^) feed rate and 30 (m*min^− 1^) cutting condition, **(a)** Al-2Cu alloy, **(b)** Al-4Cu alloy, **(c)** Al-6Cu alloy, **(d)** Al-8Cu alloy.

The wear mechanisms on the cutting tool because of cutting tests were determined using SEM image. EDS analysis was also performed on the cutting tool. SEM image and EDS analysis were performed with the help of a scanning electron microscope (SEM). Accordingly, test material adhesion and side edge wear were detected on the cutting tool. Figure 9 shows SEM images of test materials at 0.08 (mm*rev^− 1^) feed rate and 30 (m*min^− 1^) cutting condition. As a result of cutting all test materials under the same conditions, it was observed that the workpiece material adhered on the cutting tool tip and cutting edge. This can be the result of the chip adhering to the rake face as it was sliding. It was also found to cause wear on the flank edge of the cutting tools^41^. This result is similar to the study of Gomez et al.^42^.

**Fig. 10:**
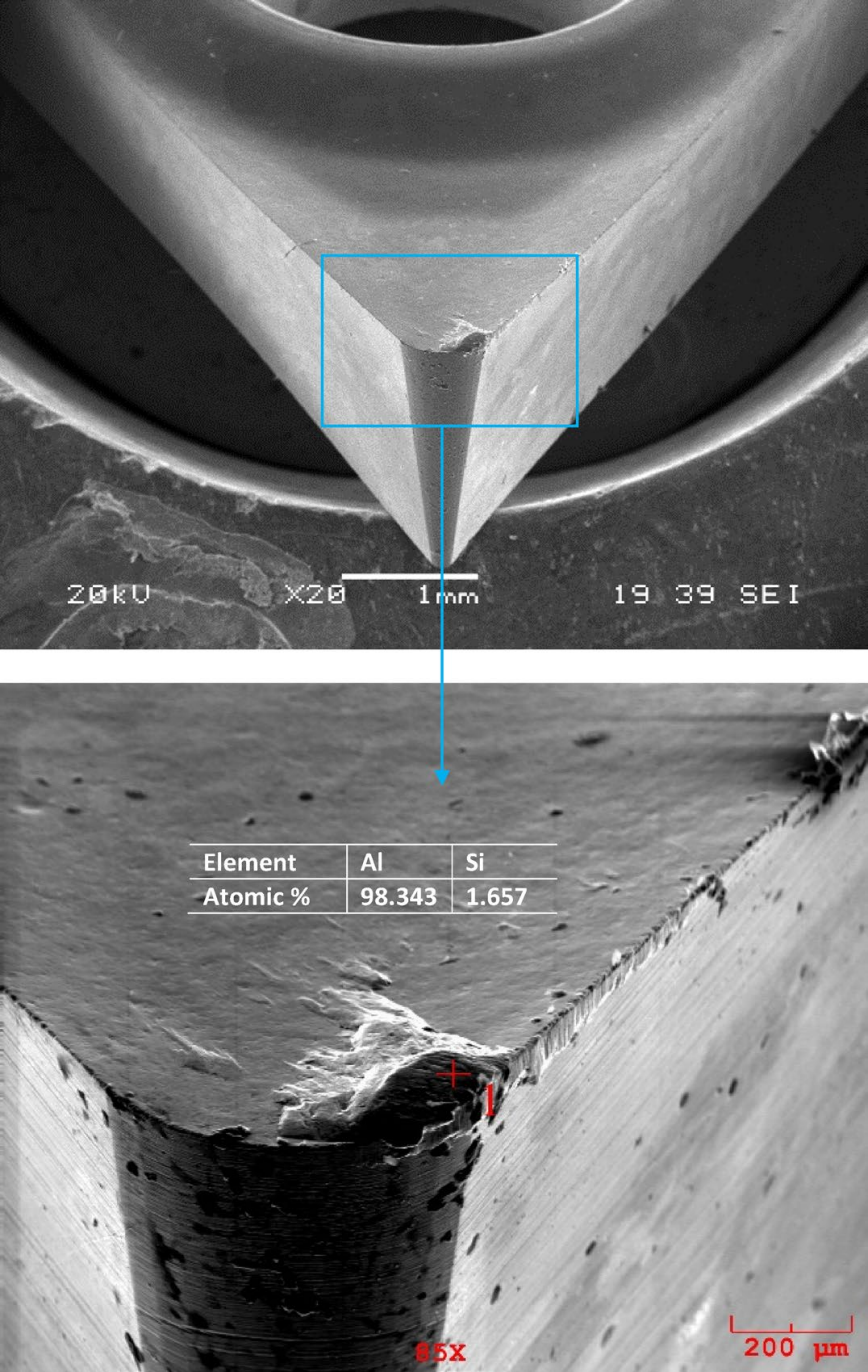
SEM image and EDS analysis of Al-4Cu alloy at 0.16 (mm*rev^-1^) feed rate and 30 (m*min^-1^) condition.

**Fig. 11:**
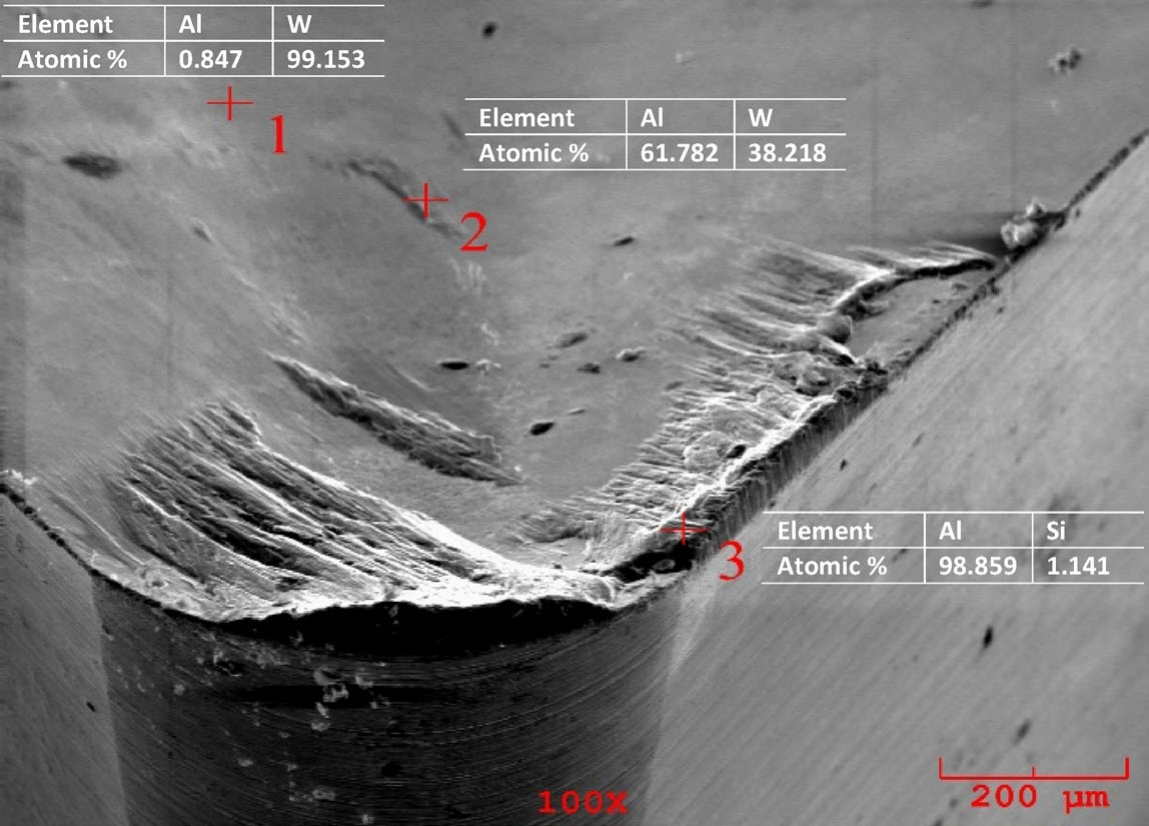
SEM image and EDS analysis of Al-6Cu alloy at 0.16 (mm*rev^-1^) feed rate and 30 (m*min^-1^) condition.

**Fig. 12:**
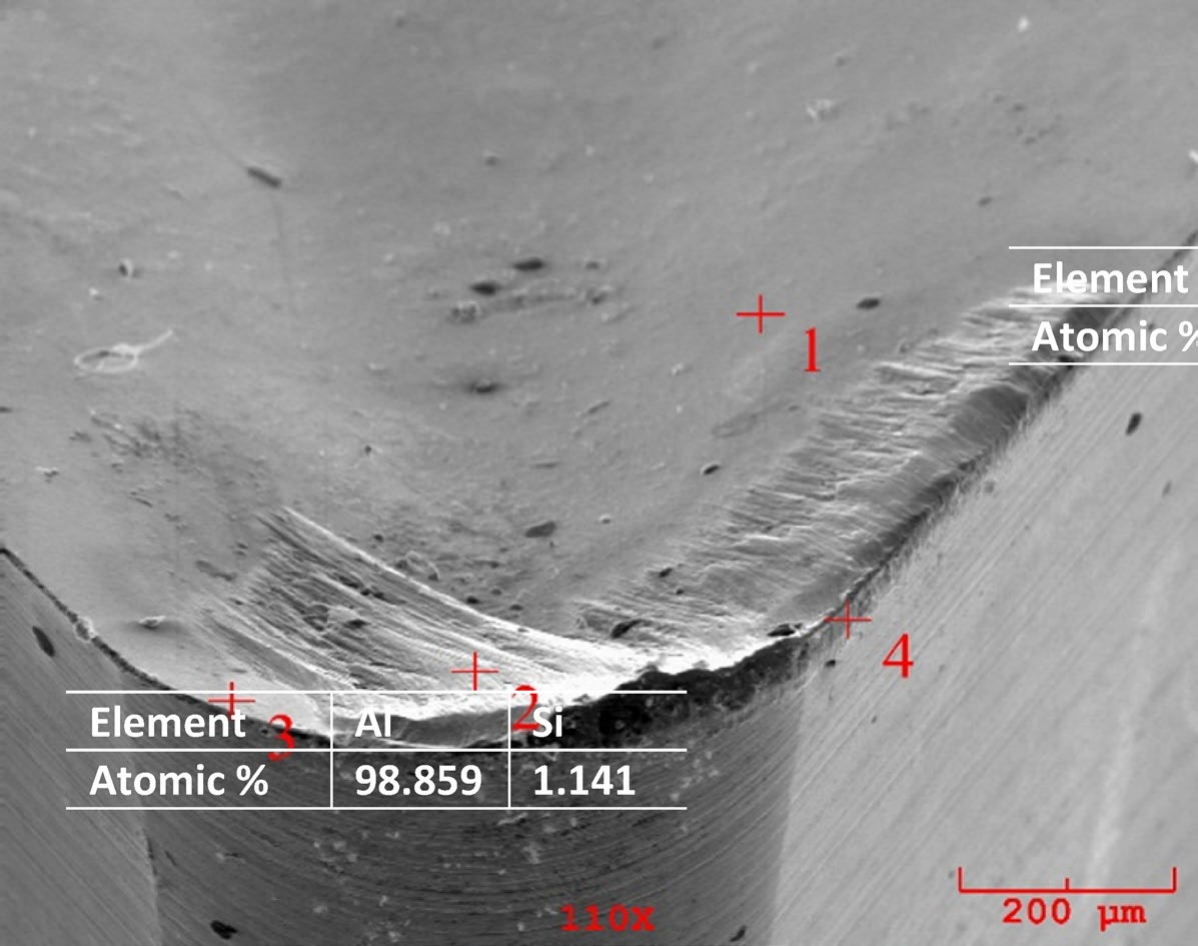
SEM image and EDS analysis of Al-8Cu alloy at 0.16 (mm*rev^-1^) feed rate and 30 (m*min^-1^) condition.

**Fig. 13:**
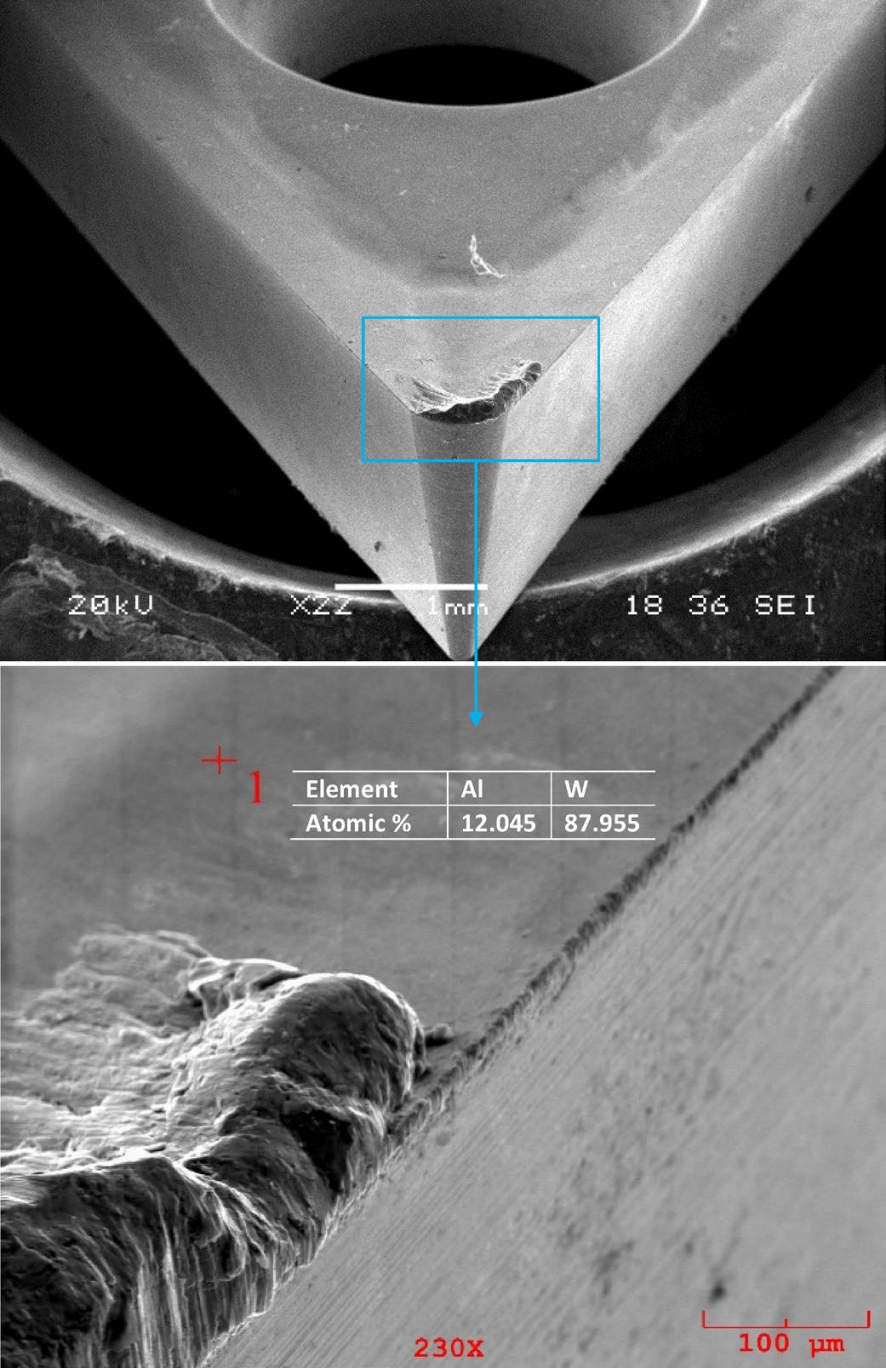
SEM image and EDS analysis of Al-2Cu alloy at 0.16 (mm*rev^-1^) feed rate and 30 (m*min^-1^) condition.

Additionally, there were severe marks where the chip moved away from the adhering layer. Different components of the workpiece material, such as silicon and aluminum, were found to be deposited because of extreme material adhesion, as was shown by the cutting tool’s EDS analysis. Workpiece material adhesion is strong when turning aluminum and aluminum alloys, leading to the observation that different workpiece migrated element components dominant at various EDS locations on the rake surface. This is observed in the high Al peaks in Figs. 10, 11 and 12. This is observed in the high Al peaks in Figs. 10, 11 and 12. In addition, W element was detected in EDS analyzes performed at other locations of the cutting tools. This element originates from the substrate of the cutting tool. Figures 10, 11 and 12 show the W elements obtained from EDS analysis. Previous studies have shown that the cutting temperature increases when v and f increase when machining Al-Cu alloy. Therefore, it shows that the formation of bonded material can be removed more quickly when heat-resistant tooling is used^42,43^. Adhesion wear is a phenomenon commonly observed during the machining of lightweight alloys, particularly aluminum. This type of wear is most prevalent within low to medium temperature ranges. Although increasing temperatures may reduce adhesion wear by promoting the flow of material over the cutting tool, excessively high temperatures can activate damage mechanisms associated with the cutting tool itself^44^. Representative SEM images and corresponding EDS analyses of the Al–2Cu alloy under a feed rate of 0.16 mm/rev and a cutting velocity of 30 m/min are shown in Fig. 13, confirming the presence of adhered workpiece material and tool substrate elements.

### Statistical analysis

The full factorial design method is a type of experimental design used to systematically investigate the effects of one or more factors on a response variable, and the interactions between these factors. This design includes all possible combinations of factor levels. For example, if there are two factors and each factor has two levels, a total of 4 trials (2 × 2) are conducted^45^. This method allows for the determination of both the independent effects of each factor (main effects) and the interactions between factors (interaction effects). This method is preferred for several reasons. Firstly, by testing all possible combinations, it provides a comprehensive understanding of the effects of the factors on the outcome. Secondly, it allows for the identification of interactions between factors. This helps to understand not only the independent effects of the factors but also how they work together. Finally, understanding the effects and interactions of factors can help in making better decisions and improving systems^46^. However, there are some disadvantages to the full factorial design method. Testing all possible combinations can be time-consuming and costly when there are many factors and levels. Additionally, as the number of factors increases, the number of trials increases exponentially. This method is particularly useful in complex systems where interactions between factors are important^47^. To clearly present all experimental combinations and the corresponding force measurements used in the full factorial analysis, the complete dataset is provided in Table 4.

**Table 3:** Full factorial machining parameter combinations and cutting-force measurements used in the statistical and ANOVA analyses.

StdOrder	RunOrder	Material type	Cutting velocity	Feed rate	Cutting force (*N*)	Feed force (*N*)
28	1	Al-6Cu	30	0.08	162.03	20.18
43	2	Al-8Cu	125	0.08	43.21	5.38
32	3	Al-6Cu	90	0.16	84.50	7.63
29	4	Al-6Cu	30	0.16	253.51	22.88
15	5	Al-2Cu	90	0.32	45.43	5.51
16	6	Al-2Cu	125	0.08	16.20	2.02
41	7	Al-8Cu	90	0.16	112.67	10.17
17	8	Al-2Cu	125	0.16	25.35	2.29
26	9	Al-4Cu	125	0.16	33.80	3.05
31	10	Al-6Cu	90	0.08	54.01	6.73
35	11	Al-6Cu	125	0.16	50.70	4.58
14	12	Al-2Cu	90	0.16	42.25	3.81
4	13	Al	90	0.08	18.00	2.24
10	14	Al-2Cu	30	0.08	81.01	10.09
40	15	Al-8Cu	90	0.08	72.01	8.97
2	16	Al	30	0.16	84.50	7.63
33	17	Al-6Cu	90	0.32	90.86	11.03
38	18	Al-8Cu	30	0.16	338.01	30.51
37	19	Al-8Cu	30	0.08	216.04	26.90
5	20	Al	90	0.16	28.17	2.54
20	21	Al-4Cu	30	0.16	169.00	15.25
3	22	Al	30	0.32	90.86	11.03
30	23	Al-6Cu	30	0.32	272.57	33.08
27	24	Al-4Cu	125	0.32	36.34	4.41
18	25	Al-2Cu	125	0.32	36.34	4.41
8	26	Al	125	0.16	16.90	1.53
21	27	Al-4Cu	30	0.32	181.71	22.05
1	28	Al	30	0.08	54.01	6.73
9	29	Al	125	0.32	18.17	2.21
34	30	Al-6Cu	125	0.08	32.41	4.04
11	31	Al-2Cu	30	0.16	126.75	11.44
23	32	Al-4Cu	90	0.16	56.33	5.08
6	33	Al	90	0.32	30.29	3.68
25	34	Al-4Cu	125	0.08	21.60	2.69
45	35	Al-8Cu	125	0.32	72.68	8.82
19	36	Al-4Cu	30	0.08	108.02	13.45
7	37	Al	125	0.08	10.80	1.35
24	38	Al-4Cu	90	0.32	60.57	7.35
36	39	Al-6Cu	125	0.32	54.51	6.62
12	40	Al-2Cu	30	0.32	136.28	16.54
39	41	Al-8Cu	30	0.32	363.42	44.10
42	42	Al-8Cu	90	0.32	121.14	14.70
22	43	Al-4Cu	90	0.08	36.01	4.48
44	44	Al-8Cu	125	0.16	67.60	6.10
13	45	Al-2Cu	90	0.08	27.00	3.36

**Fig. 14:**
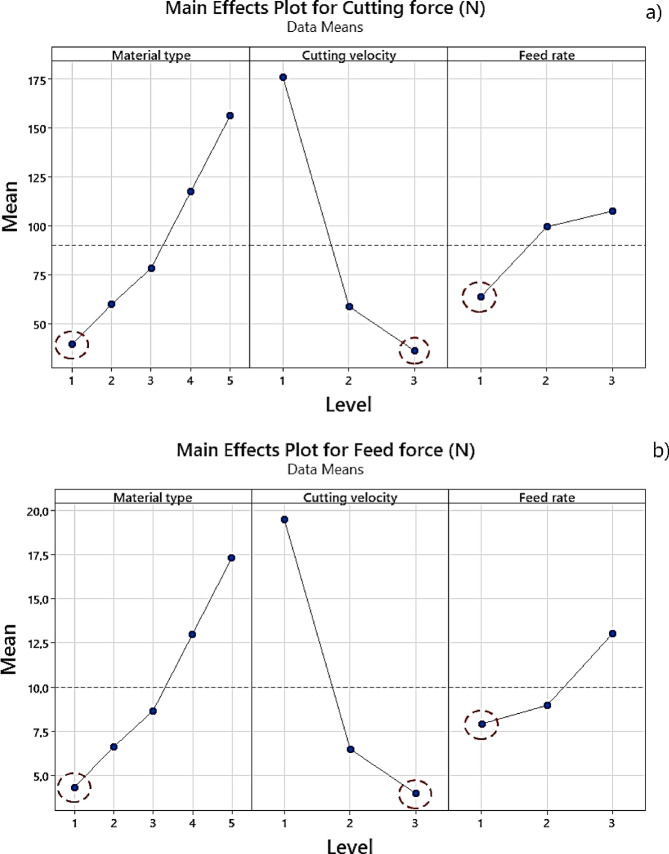
Optimum shear force conditions **(a)** Shear force, **(b)** Feed force.

The optimal cutting conditions obtained according to the full factorial design method are shown in Fig. 14 for both cutting force and feed force. Accordingly, the optimal cutting forces were achieved under the A1B3C1 conditions. This finding is consistent with many studies in the literature^48–50^.

**Fig. 15:**
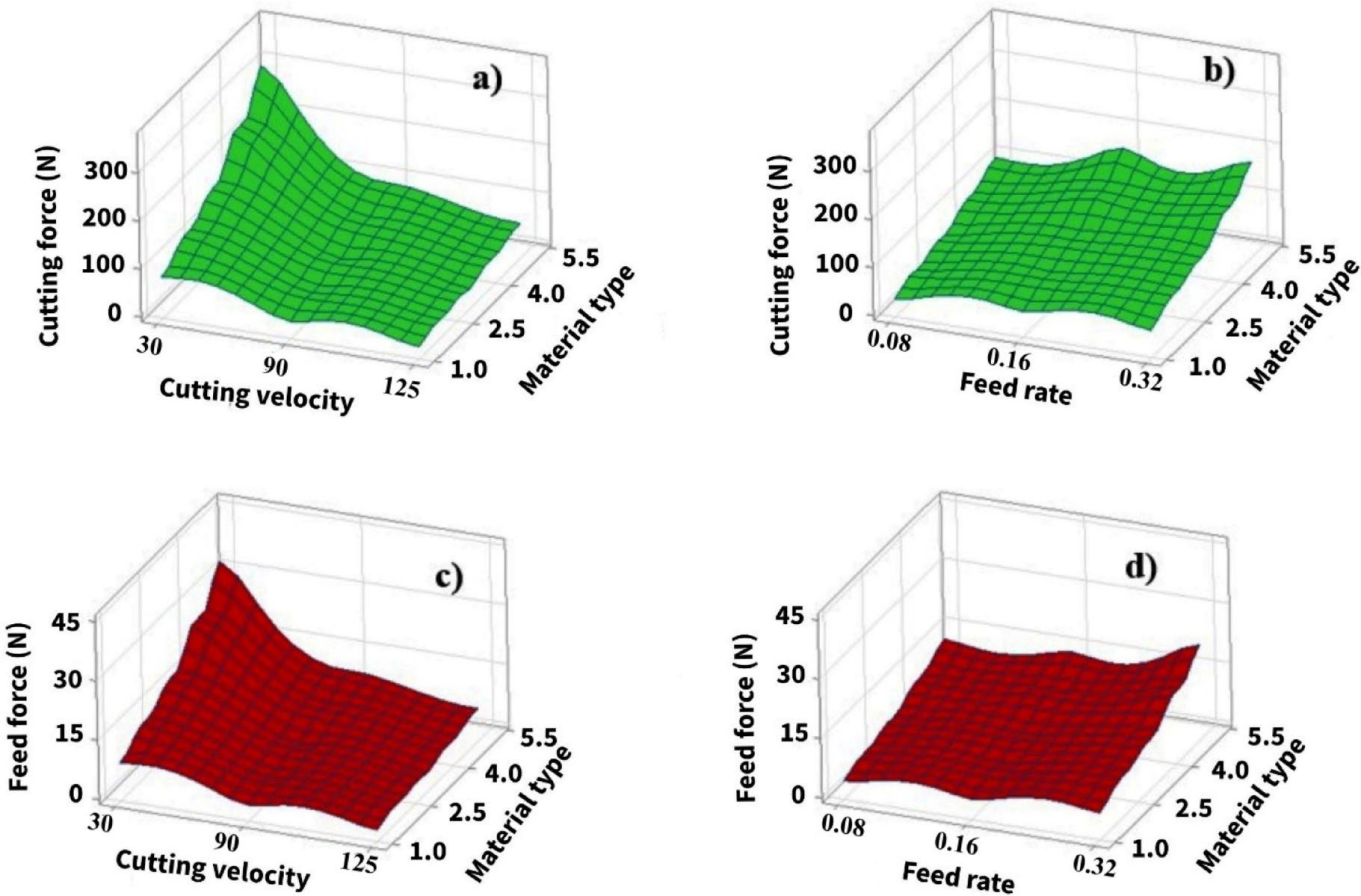
Surface plots for **(a)** cutting force, **(b)** feed force.

The interaction effects of cutting velocity and feed rate on cutting force and feed force are further illustrated using three-dimensional surface plots, as shown in Fig. 15.

ANOVA (Analysis of Variance) is a statistical method used to compare the averages of multiple groups and evaluate the differences between them. It is commonly applied in experimental studies to compare the performance of groups exposed to different conditions. The ANOVA table presents the differences between groups, sources of variance, F-test results, degrees of freedom, and the proportion of variance explained. Through this table, the effects of variables on groups and statistically significant differences can be identified. A p-value of less than 0.05 is considered indicative of statistical significance, meaning that the observed differences are unlikely to have occurred by chance.

In addition to these parameters, the ANOVA table also provides the *PC* (%) (Percentage Contribution) value, which indicates the percentage share of each factor in the total variance of the response. The ANOVA tables for both cutting force and feed force were created separately using Minitab 21 software. In these tables, factors with an F value greater than 4 and a p-value less than 0.05 are considered statistically significant^41^. Tables 5 and 6 present the ANOVA results calculated for cutting force and feed force.

Upon careful examination of these tables, it is observed that the most significant factor influencing cutting forces is cutting velocity. According to Table 5, the *PC* (%) of cutting velocity on cutting force is 53.31%, while material type contributes 24.90% and feed rate contributes 5.66%. Similarly, in Table 6, cutting velocity shows a *PC* (%) of 53.74% on feed force, followed by material type with 25.11% and feed rate with 5.20%. All statistically significant factors meet the criterion of p-value < 0.05.

**Table 4:** ANOVA of cutting force.

Source	DF	Adj SS	Adj MS	F-Value	F Table	*P*-Value	PC(%)
Material type	4	968.3	242.09	13.90^a^	2.66^a^	0.000	24.90
Cutting velocity	2	2073	1036.51	59.53	3.28^a^	0.000	53.31
Feed rate	2	220	109.98	6.32	3.28^a^	0.004	5.66
Error	36	626.8	17.41				
Total	44	3888.1					

^a^99.5% confidence level.

**Table 5:** ANOVA of feed force.

Source	DF	Adj SS	Adj MS	F-Value	F Table	*P*-Value	PC (%)
Material type	4	79,151	19,788	14.17	2.66^a^	0.000	25.11
Cutting velocity	2	169,427	84,713	60.66	3.28^a^	0.000	53.74
Feed rate	2	16,396	8198	5.87	3.28^a^	0.006	5.20
Error	36	50,273	1396				
Total	44	315,247					

^a^99.5% confidence level.

Regression analysis is a statistical technique used to model and analyze the relationship between a dependent variable and one or more independent variables. This method is primarily utilized to predict the impact of certain variables on others. Regression analysis finds applications in various fields such as economics, engineering, biology, and social sciences. In experimental studies, it aids in understanding and predicting the relationships between variables, making the data more meaningful. Additionally, it contributes to the modeling and optimization of complex systems and processes.

In this study, regression analysis was conducted separately for each material type to account for the distinct characteristics of each material, rather than treating material type as a single numerical variable. This approach ensures that the unique behavior of each material is accurately captured in the models^51^.

The mathematical equations obtained using Minitab 21 software were calculated separately for both cutting force and feed force. For each material type, the regression equations together with their R² values are given below:

- **Material type 1**.
- Cutting force (N) = 81.41 − 30.58 × Cuttting Velocity + 9.42 × Feed Rate (1).
- R² = 0.92.
- Feed force (N) = 8.89 − 3.38 × Cuttting Velocity + 1.10 × Feed Rate (2).
- R² = 0.89.
- **Material type 2**.
- Cutting force (N) = 117.06 − 44.36 × Cuttting Velocity + 15.64 × Feed Rate (3).
- R² = 0.94.
- Feed force (N) = 12.73 − 4.89 × Cuttting Velocity + 1.83 × Feed Rate (4).
- R² = 0.91.
- **Material type 3**.
- Cutting force (N) = 162.82 − 61.17 × Cuttting Velocity + 18.83 × Feed Rate (5).
- R² = 0.95.
- Feed force (N) = 17.78 − 6.77 × Cuttting Velocity + 2.20 × Feed Rate (6).
- R² = 0.92.
- **Material type 4**.
- Cutting force (N) = 244.23 − 91.75 × Cuttting Velocity + 28.25 × Feed Rate (7).
- R² = 0.96.
- Feed force (N) = 26.68 − 10.15 × Cuttting Velocity + 3.30 × Feed Rate (8).
- R² = 0.93.
- **Material type 5**.
- Cutting force (N) = 325.64 − 122.33 × Cuttting Velocity + 37.66 × Feed Rate (9).
- R² = 0.97.
- Feed force (N) = 35.57 − 13.54 × Cuttting Velocity + 4.40 × Feed Rate (10).
- R² = 0.94.

These separate equations demonstrate that cutting velocity has a significant negative effect on both cutting and feed forces, while feed rate has a positive effect. The high R² values (0.89–0.97) confirm that the models explain the experimental data well, thereby ensuring statistical reliability. In addition to the R² values, the magnitude of the regression coefficients further supports the statistical and physical significance of the models. Accordingly, since the regression coefficients obtained in both equations are greater than 0.80, these equations are both statistically and physically significant. Because for the regression coefficient to be statistically reliable, this value should be between 0.8 and 1^52^.

To verify the reliability and predictive accuracy of the regression equations obtained for each material type, a model validation study was conducted. In this validation, selected machining conditions were used to compare the experimental cutting and feed forces with the values predicted by the regression models. The comparison results are presented in Table 6.

As shown in the table, the predicted values are generally in close agreement with the experimental data, with error percentages mostly below 15%. Only in extreme parameter combinations (e.g., Al–8Cu alloy at low cutting velocity and high feed rate), the deviation between predicted and experimental values increases, which reflects the limitations of the linear regression model under boundary conditions. Overall, the validation confirms that the regression equations provide statistically reliable predictions of cutting and feed forces across the tested machining conditions.

**Table 6:** Comparison of experimental and predicted values for model validation.

Material type	Cutting velocity (m/min)	Feed rate (mm/rev)	Cutting force (Exp, *N*)	Cutting force (Pred, *N*)	Error %	Feed force (Exp, *N*	Feed force (Pred, *N*)	Error %
Al-6Cu	30	0.08	162.03	180.73	11.6%	20.18	19.83	1.7%
Al-2Cu	30	0.08	81.01	88.34	9.0%	10.09	9.67	4.2%
Al-8Cu	30	0.32	363.42	206.96	43.0%	44.10	29.63	32.8%

The findings of this study are consistent with previous investigations on machinability under MQL conditions, statistical analysis using ANOVA, and tool wear behavior. Ekinovic et al.^53^ reported that MQL turning of carbon steel St52-3 reduced cutting forces by approximately 17% compared to dry machining, emphasizing the energy-saving and sustainability benefits of MQL. Similarly, Hadad and Sadeghi^54^ demonstrated that MQL turning of AISI 4140 steel significantly lowered tool-chip interface temperatures (up to 350 °C compared to dry machining) and improved tool life, confirming the cooling and lubrication efficiency of MQL. Akincioğlu et al.^55^ applied ANOVA and regression analysis to optimize surface roughness in turning Hastelloy C22, showing that feed rate was the dominant factor influencing tool wear and surface finish. More recently, Prasad et al.^56^ highlighted that MQL reduced cutting forces (10–30%), improved surface finish (91%), and extended tool life (267%), while hybrid cryogenic–MQL approaches further enhanced sustainability. Masoudi et al.^57^ compared MQL, wet, and dry turning of AISI 1045 steel and reported that MQL significantly improved surface topography, cylindricity tolerance, and sustainability criteria relative to conventional methods. In addition, Dastgerdi et al.^58^ demonstrated through ANOVA-based statistical analysis that MQL cooling strategies reduced tool wear by 21.6% and improved surface finish by 25.3% compared to dry machining of GTD-450 stainless steel, with feed rate identified as the most influential factor for surface roughness. These comparisons validate the reliability of our ANOVA-based findings and emphasize the novelty of applying MQL to Al–Cu alloys, where cutting velocity was identified as the dominant parameter.

## Conclusion

In this study, test specimens were successfully produced using the sand mold casting method by adding copper to aluminum. The cutting behavior of these specimens was investigated on a conventional lathe utilizing the MQL technique, and the wear mechanisms on the cutting tools during turning were examined through SEM images and EDS analysis. The following key findings and conclusions were drawn from the study:

- Microstructure examinations revealed the presence of Al-Cu compounds, with their formation increasing proportionally with higher copper content. The Al-8Cu alloy exhibited the highest hardness value of 95 HB, while pure aluminum had the lowest at 34 HB.
- Cutting forces increased with higher feed rates and decreased with increasing cutting velocities for all specimens. The highest cutting forces were recorded for the Al-8Cu alloy (Ft = 216.03 N, Ff = 26.90 N), while the lowest were observed for pure aluminum (Ft = 54 N, Ff = 6.07 N).
- SEM and EDS analyses identified flank wear and adhesion as the primary wear mechanisms on the cutting tools.
- ANOVA analysis confirmed that cutting velocity was the most influential factor affecting cutting forces, followed by material type and feed rate. Regression analysis validated the critical role of cutting velocity, material type, and feed rate in influencing cutting and feed forces, and mathematical models were successfully developed.
- As potential future work, the effects of alternative cutting fluid techniques, such as cryogenic cooling or high-pressure cooling, could be explored. Additionally, the wear mechanisms and cutting performance of coated cutting tools could be examined based on various coating types.

## Data Availability

The data that support the findings of this study are available from the corresponding author upon reasonable request.
